# Syk and Src Family Kinases Regulate C-type Lectin Receptor 2 (CLEC-2)-mediated Clustering of Podoplanin and Platelet Adhesion to Lymphatic Endothelial Cells*[Fn FN2]

**DOI:** 10.1074/jbc.M114.584284

**Published:** 2014-11-03

**Authors:** Alice Y. Pollitt, Natalie S. Poulter, Eelo Gitz, Leyre Navarro-Nuñez, Ying-Jie Wang, Craig E. Hughes, Steven G. Thomas, Bernhard Nieswandt, Michael R. Douglas, Dylan M. Owen, David G. Jackson, Michael L. Dustin, Steve P. Watson

**Affiliations:** From the ‡ University of Birmingham, Centre for Cardiovascular Sciences, Institute of Biomedical Research, College of Medical and Dental Sciences, Edgbaston, Birmingham B15 2TT, United Kingdom,; the § University Medical Center Utrecht, Department of Clinical Chemistry and Haematology, 3584 CX, Utrecht, The Netherlands,; the ¶ Human Immunology Unit, Weatherall Institute of Molecular Medicine, University of Oxford, John Radcliffe Hospital, Headington, Oxford OX3 9DS, United Kingdom,; the ‖ Department of Experimental Biomedicine, University Hospital, University of Würzburg, Würzburg 97080, Germany,; the ** School of Immunity and Infection, University of Birmingham, Birmingham B15 2TT, United Kingdom,; the ‡‡ Department of Neurology, Dudley Group National Health Service Foundation Trust, Dudley DY1 2HQ, United Kingdom,; the §§ Randall Division of Cell and Molecular Biophysics, King's College London, Guy's Campus, London SE1 1UL, United Kingdom,; the ¶¶ Kennedy Institute of Rheumatology, Nuffield Department of Orthopaedics, Rheumatology and Musculoskeletal Diseases, University of Oxford, Headington OX3 7FY, United Kingdom, and; the ‖‖ Department of Molecular Pathogenesis, New York University, Skirball Institute of Biomolecular Medicine, School of Medicine, New York University Langone Medical Center, New York, New York 10016

**Keywords:** Endothelial Cell, Lipid Bilayer, Platelet, Receptor, Tyrosine-Protein Kinase (Tyrosine Kinase), CLEC-2, ITAM, Podoplanin, Src Family Kinase, Syk

## Abstract

The interaction of C-type lectin receptor 2 (CLEC-2) on platelets with Podoplanin on lymphatic endothelial cells initiates platelet signaling events that are necessary for prevention of blood-lymph mixing during development. In the present study, we show that CLEC-2 signaling via Src family and Syk tyrosine kinases promotes platelet adhesion to primary mouse lymphatic endothelial cells at low shear. Using supported lipid bilayers containing mobile Podoplanin, we further show that activation of Src and Syk in platelets promotes clustering of CLEC-2 and Podoplanin. Clusters of CLEC-2-bound Podoplanin migrate rapidly to the center of the platelet to form a single structure. Fluorescence lifetime imaging demonstrates that molecules within these clusters are within 10 nm of one another and that the clusters are disrupted by inhibition of Src and Syk family kinases. CLEC-2 clusters are also seen in platelets adhered to immobilized Podoplanin using direct stochastic optical reconstruction microscopy. These findings provide mechanistic insight by which CLEC-2 signaling promotes adhesion to Podoplanin and regulation of Podoplanin signaling, thereby contributing to lymphatic vasculature development.

## Teaser

**Background:** The interaction of platelet CLEC-2 with Podoplanin is critical for development of the lymphatics.

**Results:** CLEC-2 forms a central cluster upon engagement with Podoplanin, which clusters Podoplanin. Clustering is dependent on Syk and is critical for adhesion.

**Conclusion:** Clustering regulates the interaction of platelets with lymphatic endothelial cells.

**Significance:** These findings account for the similar lymphatic phenotype of CLEC-2- and Syk-deficient mice.

## Introduction

The C-type lectin-like receptor CLEC-2^3^ is a type II transmembrane protein that is highly expressed on the surface of platelets and megakaryocytes and less so on dendritic cells (1, 2). Unlike classical immunoreceptor tyrosine-based activation motif (ITAM)-containing receptors, which possess a tandem Y*XX*L sequence, CLEC-2 is characterized by a single Y*XX*L sequence, known as a hemITAM, in its cytosolic tail. Tyrosine phosphorylation of the hemITAM sequence following ligand engagement is mediated by the interplay between Src family and Syk tyrosine kinases (3–5). Recruitment and stimulation of Src family and Syk kinases lead to platelet activation involving the adapter protein SLP-76 and effector enzyme phospholipase Cγ2 (1, 3, 6, 7).

The only established endogenous ligand for CLEC-2 is Podoplanin. This sialomucin-like glycoprotein has a wide tissue distribution and is found at a high level in lung type I alveolar cells, kidney podocytes, choroid plexus epithelium, lymphatic endothelial cells (LECs), and fibroblastic reticular cells within secondary lymphoid organs. Podoplanin is not found on vascular endothelial cells. Podoplanin is up-regulated in a variety of tumors and on macrophages following lipopolysaccharide stimulation (8–10). Cells expressing Podoplanin or recombinant forms of the Podoplanin extracellular domain have been shown to induce platelet activation (6, 7, 9, 11).

Podoplanin has a single transmembrane region and a short cytoplasmic tail of nine amino acids (12). Three basic residues and a phosphorylated serine within the cytoplasmic tail confer direct binding of the Podoplanin cytoplasmic tail to ezrin and moesin, members of the ERM (ezrin, radixin, and moesin) family. ERM proteins provide a link between integral membrane proteins and the actin cytoskeleton. Overexpression of Podoplanin results in increased ERM protein phosphorylation (13, 14), and this interaction underlies many of the effects of Podoplanin on the actin cytoskeleton. The small GTPase RhoA is implicated in regulating the association of ERM proteins with their membrane targets. Knockdown of Podoplanin in microvascular LECs abrogates RhoA activation in association with a reduction in cell migration and tube formation (15, 16). In cancer cell lines, overexpression of Podoplanin leads to RhoA activation, and this correlates with the acquisition of a migratory phenotype and the onset of epithelial-mesenchymal transition (13). Significantly, knockdown or clustering of Podoplanin has been shown to prevent cell migration consistent with a model in which the transmembrane protein regulates the cytoskeleton through constitutive signaling (13, 17, 18).

Through an interaction with Podoplanin, CLEC-2 is essential for maintaining separation of blood and lymphatic vasculatures and preventing hemorrhaging in the brain during development (7, 11, 17, 19–23). Blood-lymphatic mixing and hemorrhaging is present in mice with a megakaryocyte/platelet-specific deletion of CLEC-2, suggesting a role for this lineage in development (17). Megakaryocyte/platelet-specific deletion of the CLEC-2 signaling proteins, Syk and SLP-76, also results in blood-lymphatic mixing and brain hemorrhaging (17, 21, 24). These observations indicate that the direct interaction of CLEC-2 with Podoplanin and the initiation of platelet signaling are critical for normal development. Consistent with this, platelets are activated upon binding to LECs as shown by imaging of Ca^2+^. Elevation of Ca^2+^ was blocked in the presence of a Syk inhibitor (25).

In this study, we have investigated the mechanism by which platelets and primary LECs and Podoplanin-expressing cell lines interact. We have monitored the dynamics of CLEC-2-Podoplanin interactions and investigated the role of CLEC-2 signaling in the interaction using a planar lipid bilayer system that maintains lateral mobility of recombinant Podoplanin. This method has been used to study the immunological synapse formed by T, B, and natural killer cells (26). Using glass-supported planar lipid bilayers containing recombinant Podoplanin, we demonstrate that CLEC-2-mediated signaling enhances platelet adhesion by regulating formation of Podoplanin clusters. We show that adhesion of platelets to primary mouse LECs at low shear is regulated by Src family and Syk tyrosine kinases. These findings provide a mechanism for the stable adhesion of platelets to Podoplanin-expressing cells and also the regulation of Podoplanin signaling, which likely contributes to the requirement for CLEC-2 signaling in development of the lymphatic system.

## EXPERIMENTAL PROCEDURES

### 

#### 

##### Reagents and Antibodies

Monobiotinylation of mPDPN-Fc and 17D9 was performed as previously described for the monobiotinylation of antibodies (27). Alexa 488 and Dylight 595 labeling of monobiotinylated mPDPN-Fc was performed by following the manufacturer's instructions (Invitrogen and Thermo Scientific). Phycoerythrin-conjugated anti-human Podoplanin NZ-1–3 and rat IgG2a isotype control (eBioscience). Quantum Simply Cellular anti-rat IgG microspheres (Bangs Laboratories, Inc). Lotrafiban (GlaxoSmithKline), and dasatinib (LC Laboratories) were used. PRT-060318 was kindly donated by Portola Pharmaceuticals. Antibodies against lymphatic endothelial cell markers Prox-1 and LYVE-1 were purchased from Fitzgerald Industries and R&D Systems, respectively. mCLEC-2 antibodies INU1 and 17D9 have been previously described (28, 29). All other reagents were purchased from Sigma-Aldrich.

##### Expression of mPDPN-Fc

The extracellular domain of mouse Podoplanin was amplified from cDNA generated from a C57BL/6 kidney with the primers mPodoHindFor (GATCAAGCTTATGTGGACCGTGCCAGTGTTG) and mPodoFcRev (GATCGGATCCACTTACCTGTCAGGGTGACTACTGGCAAGCC). After digestion with HindIII and BamHI, the PCR product was cloned into IgFc vector pcDNA3Ig to yield a construct encoding the extracellular domain of Podoplanin fused at the C terminus to the Fc region of human IgG1_._ For expression and purification of the Podoplanin-Fc fusion protein, the expression vector was transfected into 293T cells using the polyethylenimine transfection method. The fusion protein was purified by affinity chromatography using protein A-Sepharose. mPDPN-Fc-containing fractions were dialyzed into PBS.

##### mPodoplanin Constructs

Full-length mouse Podoplanin was cloned into pAcGFPN1 and pDsRedmonomerN1 (Clontech).

##### mCLEC-2 GFP Construct

Full-length mCLEC-2 was cloned into pAcGFPC1 to generate an N-terminal GFP fusion of mCLEC-2. An 12-amino acid linker was added between the GFP and mCLEC-2 to maintain function. Receptor function was assessed using an NFAT luciferase reporter assay (30) (data not shown).

##### Mouse Models

Generation and characterization of a conditional SykR41A knock-in mouse (PF4-Cre sykR41A^fl/fl^) are described elsewhere (64). PF4-Cre clec1b^fl/fl^ and Lifeact-GFP transgenic mice have been described previously (17, 31). The mice were housed in accordance with institutional guidelines approved by the United Kingdom Home Office.

##### Preparation of Mouse Platelets

Whole blood was drawn from the inferior vena cava into acid citrate dextrose (1/9 v/v) from CO_2_-asphyxiated mice following isofluorane anesthesia. Washed mouse platelets were obtained as previously described (32). Briefly, platelets were obtained by centrifugation using prostaglandin I_2_ to prevent activation during the isolation procedure. Washed platelets were then resuspended in modified HEPES Tyrode buffer (134 mm NaCl, 2.9 mm KCl, 12 mm NaHCO_3_, 0.34 mm NaH_2_PO_4_, 1 mm MgCl_2_, 5.5 mm glucose, 1 mm MgCl_2_, 20 mm HEPES, and 5 mm glucose, pH 7.3) at a density of 2 × 10^7^ platelets/ml.

##### Static Adhesion Assays

Acid-washed coverslips were incubated with a 10 μg/ml suspension of mPDPN-Fc overnight at 4 °C. Surfaces were then blocked with denatured BSA (5 mg/ml) for 1 h at room temperature followed by subsequent washing with PBS before use in spreading assays. Platelets (2 × 10^7^/ml) were allowed to adhere for 45 min at 37 °C. Nonadhered platelets were removed by PBS washes before fixation with 10% formalin, neutral buffered, for 10 min at room temperature.

##### Cell Adhesion Assays

Cells were transfected with the indicated constructs 48 h before the addition of platelets. Platelets were allowed to adhere for 1 h at 37 °C. Nonadhered platelets were removed by PBS washes prior to fixation with 10% formalin, neutral buffered, for 10 min at room temperature.

##### Mouse Lymphatic Endothelial Cell Preparation

Primary mouse dermal LECs were isolated from C57BL/6 P2-3 pups. Briefly, the epidermis was removed from the dermis following an overnight incubation at 4 °C with 2 mg/ml dispase (Invitrogen). The tissue was then digested using 2 mg/ml collagenase A while shaking at 37 °C for 30 min. A single cell suspension was then added to 0.1% gelatin-coated tissue culture flasks in RPMI medium supplemented with 10% fetal calf serum, 1% penicillin/streptomycin, and glutamine. Nonadhered cells were removed after 3 h, and adhered cells were then cultured in freshly made endothelial cell growth medium (EGM-2; Lonza). Following an overnight incubation at 37 °C, adhered cells were dislodged using Accutase (Sigma-Aldrich). LECs were isolated with anti-LYVE-1 antibody-conjugated magnetic beads and grown in a 0.1% gelatin-coated tissue culture flask with supplemented EGM-2. The cells were seeded into 8-well chamber culture slides (BD Falcon) or Ibidi μ-slides VI 0.4 (Thistle Scientific) until a confluent monolayer of LECs had formed.

##### Flow Adhesion Assays

Blood was drawn as previously described into sodium-heparin (5 units/ml) and PPACK (40 μm) to prevent thrombin generation. Blood was perfused through the Ibidi channels for 4 min at a shear rate of 50 s^−1^ at 37 °C. Nonadhered platelets were removed by a Tyrode's buffer wash prior to fixation with 10% formalin, neutral buffered, for 10 min at room temperature. The integrity of the mouse LEC monolayer was confirmed with LYVE-1 staining, and platelets were stained with an anti-αIIb antibody.

##### Preparation of Planar Lipid Bilayers

Planar lipid bilayers were generated as previously described (33). Liposomes containing biotinylated lipids (cap-biotinyl-phosphatidylethanolamine) were mixed with 1,2-dioleoylphophatidylcholine liposomes (Avanti Polar Lipids, Inc.). Liposomes were deposited on a glass coverslip cleaned by piranha solution (a mixture of sulfuric acid and hydrogen peroxide) assembled in Bioptechs FCS2 chambers. Bilayers were blocked with PBS, 2% fatty acid-free BSA (Sigma). Monobiotinylated mPDPN-Fc or monobiotinylated mCLEC-2 antibody (17D9) was loaded into the bilayers following a previous incubation with Alexa Fluor 405 streptavidin. Bilayer mobility was confirmed using fluorescence recovery after photon bleaching. A cytometric method was used to estimate the number of mPDPN-Fc molecules incorporated into a bilayer. Bilayers containing Alexa 488-conjugated mPDPN-Fc were reconstituted on glass beads and compared with standard values obtained from microbeads with different calibrated Alexa 488 fluorophores on their surface (Bangs Laboratories, Inc). Beads were analyzed using a FACScalibur cytometer (BD Bioscience).

##### Microscopy and Image Analysis

Platelets interacting with LECs were imaged using an integrin αIIb antibody. Platelet coverage was determined by the integrin αIIb threshold area using ImageJ and presented as a percentage of total image area. Platelet adhesion and spreading morphology were imaged using Köhler illuminated Nomarski differential interference contrast optics with a Zeiss 63× oil immersion 1.40 NA plan-apochromat lens on a Zeiss Axiovert 200M microscope. Digital images were captured by a Hamamatsu Orca 285 cooled digital camera (Cairn Research, Kent, UK) using Slidebook 4.0 (Intelligent Imaging Innovations, Inc., Denver, CO). The degree of platelet adhesion and surface area of spread platelet were calculated using ImageJ by outlining each individual platelet. The average platelet surface area of at least 50 platelets was calculated for each condition of each experimental replicate. Ten fields of view per condition of each experimental replicate were used to calculate the average number of platelets adhered per μm^2^.

All cell incubation and live cell imaging were performed at 37 °C. The contact of platelets with the planar lipid bilayer was visualized using total internal reflection fluorescence (TIRF) microscopy. The sequences in supplemental Movies S1 and S2 are representative of at least three independent experiments with at least 10 cells observed in each. Images were acquired using a Nikon TIRF system on a Nikon Eclipse Ti inverted microscope using illumination through the objective with a CFL Plan Apo 60×, 1.49 NA TIRF objective and analyzed using NIS Elements software and ImageJ. Interference reflection microscopy (IRM) and confocal images were acquired using a DM IRE2 Leica microscope using a 63× oil immersion objective. The average threshold area of 20 platelets was calculated for each condition of each experimental replicate.

##### Analysis of Podoplanin Clustering by FRET-FLIM

Clustering of Podoplanin was analyzed by FRET using time-gated FLIM, as described previously (34). In short, the fluorescence lifetimes of the donor fluorophore (488 mPDPN-Fc) were determined in the absence and presence of acceptor fluorophore (594 mPDPN-Fc) and subsequently used to calculate the FRET efficiency, defined as follows,

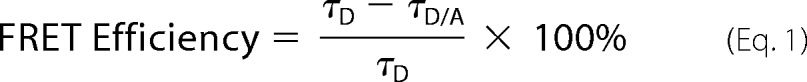
 where τ is the donor fluorophore lifetime in nanoseconds in the absence (τ_D_) and presence (τ_D/A_) of the acceptor fluorophore. To determine variation in FRET efficiency, the lifetimes of three randomly chosen quadrants were quantified.

##### Statistical Analysis

Comparisons between groups were analyzed by Student's two-tailed paired *t* test with a significance level of *p* < 0.05. Where indicated, the data were analyzed by analysis of variance test.

##### Stochastic Optic Reconstruction Microscopy

Wild type mouse platelets were spread for 45 min on 10 μg/ml Fc-Podoplanin-coated coverslips. Platelets were fixed, permeabilized, and CLEC-2-labeled using 5 μg/ml INU1 antibody. They were then secondarily labeled using an Alexa 647-conjugated goat α-rat antibody. Samples were imaged in direct stochastic optical reconstruction microscopy (dSTORM) mode using a 100 × 1.49 NA TIRF objective on a Nikon N-STORM system consisting of a Ti-E stand with Perfect Focus, Agilent MLC400 high power laser bed (647-nm laser line) and Andor iXon Ultra DU-897U EMCCD camera. To induce fluorophore blinking the samples were imaged in a PBS buffer containing 100 mm mercaptoethylamine-HCl, 50 μg/ml glucose oxidase, and 1 μg/ml catalase as detailed (35). 30,000 frames were captured using NIS Elements 4.2 with an exposure time of 9.2 ms, gain 300, and conversion gain 3. dSTORM images were reconstructed using the default settings in the Nikon STORM analysis module v3.2. Samples were drift corrected and rendered using Gaussian rendering. Cluster analysis was performed with MATLAB using a custom made algorithm. Cluster maps of the localized molecules were generated by evaluating the number of localizations within a distance, 50 nm, of each point on a 5-nm resolution grid across the region of interest. The cluster level (*L*(50)) at each of these points, *i*, was calculated as follows,

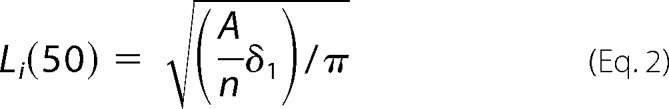
 where *A* is the area of the region of interest (in this case 3000 × 3000 nm), *n* is the total number of localizations within that area and δ_i_ is the number of localizations with a distance of 50 nm of grid point *i*. This grid was then pseudocolored by *L*(50) level to generate the cluster heat map. Edge effects were corrected by processing a border of width (*r*) around each region. At least 20 platelets from three independent experiments were analyzed.

To generate Ripley's K-function curves, the Excel plugin SpPack was used (36). Here, the cluster level (*L*(*r*)), was calculated for each region of interest for a range of *r* as follows,

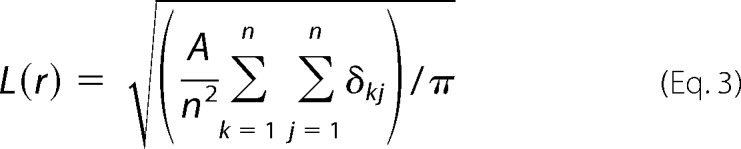
 where δ_kj_ = 1 is the distance between points *k* and *j*, is less than *r*, and is otherwise 0. In this case, the completely spatially random distribution has *L*(*r*) = *r* for all *r* and therefore has *L*(*r*) − *r* = 0. Therefore, clustered distributions have values of *L*(*r*) − *r* > 0. Border correction was performed by weighting the *L*(*r*) calculation for molecules within distance *r* of the border. To calculate 99% confidence interval for clustering, 100 completely spatially random distributions were simulated per analyzed region.

## RESULTS

### 

#### 

##### Platelet Signaling Enhances Platelet Adhesion to Primary Mouse Lymphatic Endothelial Cells under Static and Flow Conditions

**FIGURE 1.**
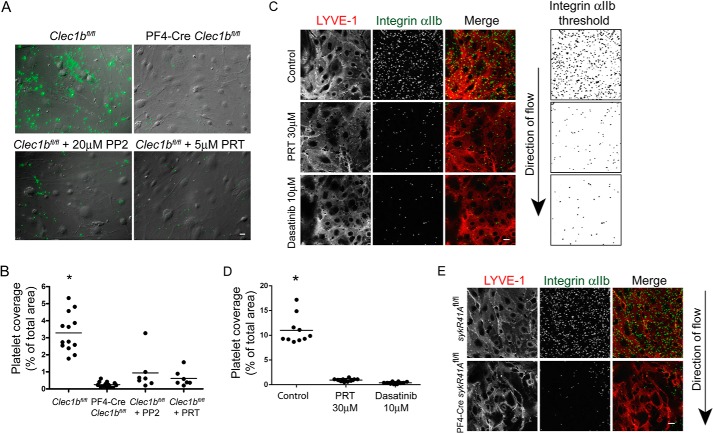
**CLEC-2 signaling supports platelet adhesion to primary mouse lymphatic endothelial cells under static and flow conditions.** *A*, CLEC-2-deficient (PF4-Cre *Clec1b*^fl/fl^) or control (*Clec1b*^fl/fl^) platelets in the presence or absence of inhibitors to Src family (PP2, 20 μm) or Syk (PRT-060318, 5 μm) kinases were incubated with a confluent monolayer of primary mouse LECs (DIC image) for 1 h at 37 °C. Platelets were identified using and anti-αIIb antibody (*green*). *Scale bar*, 20 μm. *B*, quantification of CLEC-2-deficient (PF4-Cre*Clec1b*^fl/fl^) or control (*Clec1b*^fl/fl^) platelet coverage in the presence or absence of inhibitors to Src family (PP2, 20 μm) or Syk (PRT-060318, 5 μm) kinases. *, *p* < 0.01 in analysis of variance. *C*, control blood in the presence or absence of inhibitors to Src family (10 μm dasatinib) or Syk (PRT-060318, 30 μm) kinases was perfused over primary mouse LECs at a wall shear rate of 50 s^−1^ at 37 °C. An *arrow* indicates the direction of flow. LECs and platelets were identified using anti-LYVE-1 and anti-αIIb antibodies, respectively. Integrin αIIb antibody threshold (*right panels*). *Scale bar*, 20 μm. *D*, quantification of platelet adhesion to primary mouse LECs under flow conditions in the presence or absence of inhibitors to Src family kinases (10 μm dasatinib) or Syk kinase (PRT-060318, 30 μm). *E*, control (*SykR41A* ^fl/fl^) or SykR41A expressing (PF4-Cre*SykR41A*^fl/fl^) mouse blood was perfused over primary mouse LECs at a wall shear rate of 50 s^−1^ at 37 °C. An *arrow* indicates the direction of flow. LECs and platelets were identified using anti-LYVE-1 and anti-αIIb antibodies, respectively. *Scale bar*, 20 μm. *, *p* < 0.01 in analysis of variance.

To determine the role that platelet signaling plays in the adhesion of mouse platelets to Podoplanin-expressing cells, we investigated the interaction of platelets with primary mouse dermal LECs. Prox-1 and LYVE-1 are used as a marker for LECs. This combination was used to verify the purity of mouse primary LEC preparations isolated from skin (data not shown). Platelets, in the presence and absence of Src family and Syk kinase inhibitors, were allowed to interact with a confluent monolayer of primary mouse LECs for 60 min (Fig. 1*A*). Platelets were identified with an integrin subunit αIIb antibody, and surface coverage was determined (Fig. 1*B*). Mouse platelets adhere to mouse LECs in a CLEC-2-dependent manner, because CLEC-2 deficient platelets do not adhere to primary LECs (Fig. 1). The adhesion of platelets to mouse LECs was decreased by over 70% in the presence of the selective tyrosine kinase inhibitors PP2 (Src family) and PRT-060318 (Syk). Although an effect of PP2 on the LECs cannot be excluded because of the ubiquitous expression of Src family kinases, the effect of the highly selective Syk inhibitor PRT-060318 (37) on these cells is likely to be minimal because Syk has a hematopoietic cell-specific expression. Lineage tracking has demonstrated that Syk is not expressed in lymphatic or blood endothelial cells in mice (38). We confirmed the absence of Syk from mLECs by immunoblotting for Syk (data not shown). Moreover, the same concentration of PRT-060318 abolished platelet Syk-dependent collagen signaling but not Src kinase-mediated phosphorylation of the GPVI-FcR γ-chain complex,^4^ demonstrating its specificity.

Given that the interaction between platelets and LECs are expected to occur under conditions of venous flow, mouse blood was perfused over a confluent monolayer of primary mouse LECs at a wall shear rate of 50 s^−1^ (Fig. 1*C*). To exclusively investigate platelet-endothelial interactions, perfusion was performed in the presence of the αIIbβ3 blocker lotrafiban. Platelets were identified with an integrin subunit αIIb antibody. A LYVE-1 antibody was used to ensure that the integrity of the LEC monolayer was preserved in the presence of flow. Inhibition of platelet adhesion to primary LECs was seen under flow conditions in the presence of the plasma available Src family inhibitor dasatinib or Syk inhibitor PRT-060318 (Fig. 1*D*). These results demonstrate a role for Syk and Src family kinases in supporting adhesion to endogenous Podoplanin in mouse primary LECs under static and flow conditions. To validate the role of Syk in CLEC-2-mediated adhesion to mLECs in the presence of flow, we perfused blood from a novel knockin floxed mouse in which arginine 41 in the SH2 domain of Syk has been replaced with alanine (SykR41A). This mutation renders the SH2 domain unable to bind to phosphorylated tyrosine (39). As seen with whole blood treated with a Syk inhibitor, platelet adhesion to a mLEC monolayer was reduced in platelets expressing SykR41A (PF4-Cre*SykR41A*^fl/fl^) when compared with platelets expressing wild type Syk (*SykR41A*^fl/fl^) (Fig. 1*E*).

##### Immobilized Podoplanin Supports Platelet Adhesion and Spreading through Src and Syk Kinases

**FIGURE 2.**
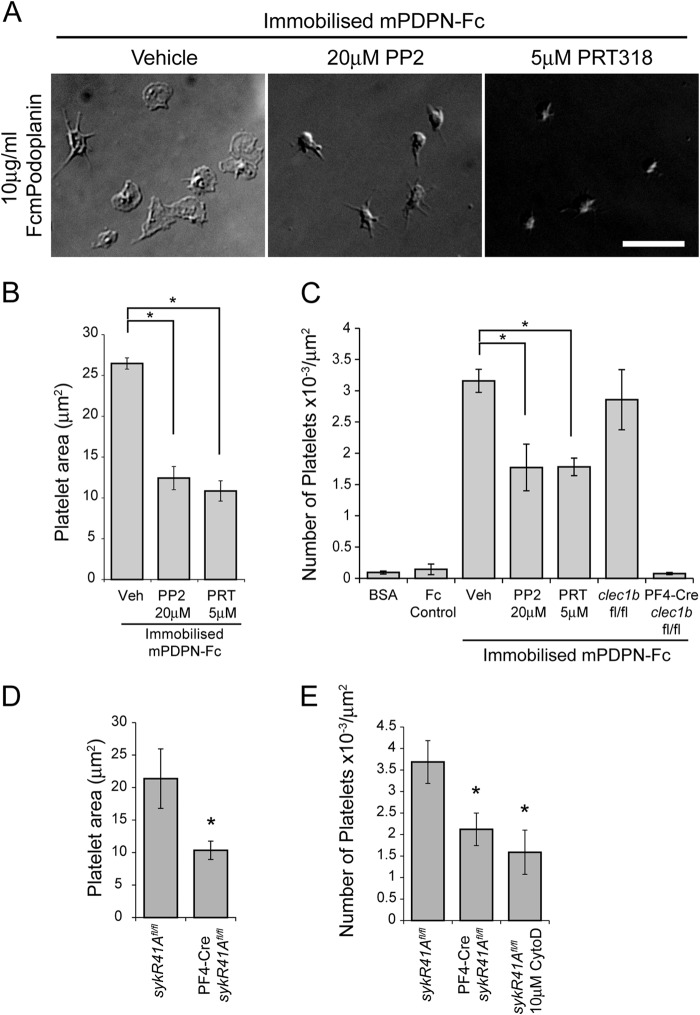
**Syk and Src family kinases support platelet adhesion to immobilized Podoplanin.** *A*, washed platelets in the presence or absence of Src family (PP2, 20 μm) or Syk (PRT-060318, 5 μm) kinase inhibitors were allowed to interact with mPDPN-Fc (10 μg/ml)-coated glass coverslips for 45 min at 37 °C and imaged using DIC microscopy. *Scale bar*, 10 μm. *B*, quantification of platelet area when interacting with immobilized mPDPN-Fc in the presence or absence of inhibitors to Src family (PP2, 20 μm) or Syk (PRT-060318, 5 μm) kinases. *C*, quantification of the number of adherent platelets interacting with BSA, a control Fc protein, or immobilized mPDPN-Fc with or without Src family (PP2, 20 μm) or Syk (PRT-060318, 5 μm) kinase inhibitors. Quantification of the number of CLEC-2-deficient (PF4-Cre *Clec1b*^fl/fl^) or control (*Clec1b*^fl/fl^) platelets interacting with mPDPN-Fc. *D*, quantification of control (*SykR41A* ^fl/fl^) or SykR41A expressing (PF4-Cre*SykR41A*^fl/fl^) platelet area when interacting with immobilized mPDPN-Fc. *E*, quantification of the number of adhered SykR41A expressing (PF4-Cre*SykR41A*^fl/fl^) or control (*SykR4A* ^fl/fl^) platelets with or without 10 μm cytochalasin D interacting with immobilized mPDPN-Fc. Images and results are the averages of three independent experiments ± S.D. *, *p* < 0.05. *Veh*, vehicle.

To investigate the interaction of platelets with Podoplanin in the absence of other potential interacting proteins on LECs, we examined the interaction of platelets with immobilized Podoplanin by allowing mouse platelets to adhere and spread on an immobilized monolayer of mPDPN-Fc, a recombinant Fc fusion of the mouse Podoplanin extracellular domain. After fixation, differential interference contrast (DIC) microscopy was used to visualize the extent of platelet spreading. Control platelets generate filopodia and lamellipodia over immobilized mPDPN-Fc reaching a size of 27.1 ± 0.7 μm^2^ (Fig. 2, *A* and *B*). This interaction was mediated by CLEC-2, because CLEC-2-deficient platelets displayed a similar level of adhesion as seen for wild type platelets on BSA or an Fc-control protein (Fig. 2*C*). Inhibition of CLEC-2 signaling by pretreating platelets with the Syk inhibitor PRT-060318 or the Src family inhibitor PP2 prevented formation of lamellipodia and markedly reduced the spread area to 13.5 ± 1.4 and 11.3 ± 1.2 μm^2^, respectively. However, there was evidence of filopodia formation in the presence of these inhibitors (Fig. 2*A*). Strikingly, Syk or Src family kinase inhibition led to a decrease of approximately 50% in the total number of platelets adhered to immobilized Podoplanin, demonstrating a critical role for platelet activation in mediating stable adhesion (Fig. 2, *A* and *C*).

To validate the role of Syk in Podoplanin-mediated platelet spreading, we used platelets expressing SykR41A. As seen with platelets treated with a Syk inhibitor, Podoplanin-mediated platelet spreading was abolished in platelets expressing SykR41A (PF4-Cre*SykR41A*^fl/fl^) when compared with platelets expressing wild type Syk (*SykR41A*^fl/fl^) (Fig. 2*D*). Platelets expressing SykR41A also had a reduction of approximately 50% in adhesion to an immobilized layer of mPDPN-Fc (Fig. 2*E*). These results demonstrate that CLEC-2-mediated activation of Src and Syk tyrosine kinases is critical for spreading and is required for efficient adhesion to an immobilized monolayer of Podoplanin. We have previously reported that activation of Syk by CLEC-2 is dependent on actin polymerization (6). Thus, it appears that CLEC-2 activation is regulated by actin-dependent feedback mechanisms. Consistent with this, the interaction of platelets with the immobilized Podoplanin is significantly reduced by disruption of the cytoskeleton (Fig. 2*E*).

##### Mobile Podoplanin Supports Platelet Adhesion and Spreading through Src and Syk Kinases

**FIGURE 3.**
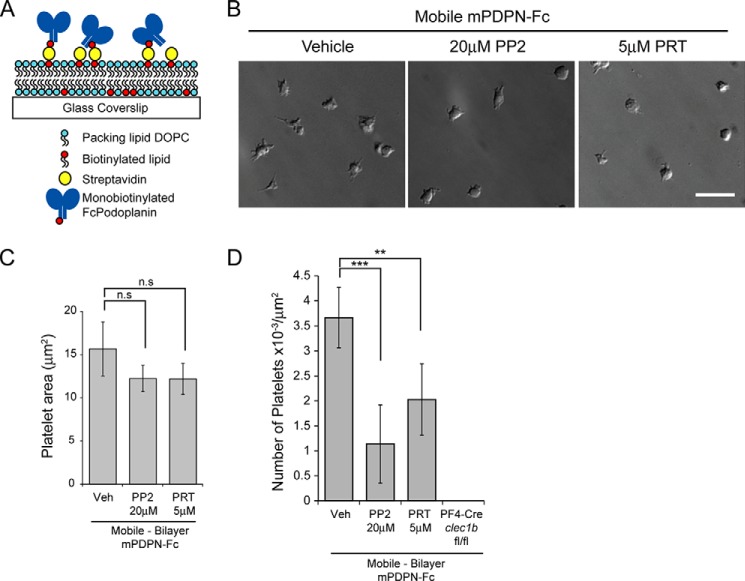
**Syk and Src family kinases support platelet adhesion to mobile Podoplanin.** *A*, schematic of the experimental system for mobile Podoplanin. Platelets were allowed to interact with glass-supported planar lipid bilayers containing biotinylated lipids, to which monobiotinylated recombinant Podoplanin (mPDPN-Fc) was tethered via streptavidin. *DOPC*, dioleoylphosphocholine. *B*, washed platelets pretreated with or without Src family (PP2, 20 μm) or Syk (PRT-060318, 5 μm) kinase inhibitors were allowed to interact with glass-supported planar lipid bilayers containing mobile mPDPN-Fc for 45 min at 37 °C and imaged using DIC microscopy. *Scale bar*, 10 μm. *C*, quantification of platelet area when interacting with mobile mPDPN-Fc with or without inhibitors to Src family (PP2, 20 μm) or Syk (PRT-060318, 5 μm) kinases. The results are the averages of four independent experiments ± S.D. *D*, quantification of the number of adherent platelets interacting with mobile mPDPN-Fc with or without inhibitors to Src family (PP2, 20 μm) or Syk (PRT-060318, 5 μm) kinases. Images and results are the averages of three independent experiments ± S.D. **, *p* < 0.01; ***, *p* < 0.001. *n.s.*, not significant; *Veh*, vehicle.

The results in Fig. 1 demonstrate a critical role for CLEC-2 signaling in supporting adhesion to membrane-bound endogenous Podoplanin. To investigate the molecular mechanisms governing the role of CLEC-2, Src family and Syk kinases in platelet adhesion to membrane Podoplanin, we modeled the situation using a planar lipid bilayer system. To achieve this, we used glass-supported lipid bilayers containing biotinylated lipids to which monobiotinylated mPDPN-Fc was tethered via streptavidin (Fig. 3*A*).

We sought to introduce Podoplanin into the supported lipid bilayers at a level similar to that in Podoplanin-expressing cells. The mean surface density of Podoplanin on HEK293T cells, human dermal microvascular endothelial (HMEC-1) cells, and squamous cell carcinoma (FaDu) cells was determined by comparing Podoplanin antibody-stained cells to calibration beads with known antigen binding capacities (Table 1). Glass beads, coated with mPDPN-Fc reconstituted supported lipid bilayers, were compared with standard beads of known fluorophore number to estimate the number of mPDPN-Fc molecules incorporated into the bilayer. The bilayers contained 48 ± 10 molecules mPDPN-Fc/μm^2^. Allowing for the fact that mPDPN-Fc contains two extracellular domains of Podoplanin, this falls within the 5-fold range of the density of Podoplanin on the above cell lines (Table 1).

**TABLE 1:** **Quantification of Podoplanin cell surface density**

Cell type	Podoplanin molecules/μm^2^
HEK293T	270 ± 55
HMEC-1	253 ± 30
FaDu	501 ± 42

Platelets were found to interact with the supported lipid bilayers when mPDPN-Fc is tethered to the bilayer. Omission of the streptavidin, biotinylated lipids, or monobiotinylated Podoplanin from the experimental setup results in very few platelets interacting with the lipid bilayers (data not shown). CLEC-2-deficient platelets did not interact with mPDPN-Fc-containing supported lipid bilayers, demonstrating that the interaction of platelets with the bilayer was mediated via the interaction of CLEC-2 and Podoplanin (Fig. 3*D*). DIC microscopy was used to visualize the extent of platelet spreading on mobile Podoplanin (Fig. 3*B*). Platelets underwent limited spreading on the mPDPN-Fc containing supported lipid bilayers (15.7 ± 3.1 μm^2^) in comparison with an immobilized surface of mPDPN-Fc (Figs. 2*B* and 3*C*). Platelet anchorage mediated by firm attachment to the immobilized surface of mPDPN-Fc is likely to account for the difference in spreading. Platelets treated with Src family and Syk kinase inhibitors have a similar platelet area to control platelets when allowed to interact with supported lipid bilayer-bound mPDPN-Fc (Fig. 3*C*). A decrease in platelet adhesion was seen in the presence of Src family and Syk kinase inhibitors on the supported lipid bilayers similar to that observed on immobilized mPDPN-Fc (Fig. 3*D*). These results therefore demonstrate that the role of Src family and Syk kinases in supporting adhesion to Podoplanin is not solely dependent on their role in mediating platelet spreading.

##### Inhibition of Src Family Kinases and Syk Results in Reduced Contact Area between Platelets and Lipid Bilayer Supported Podoplanin

**FIGURE 4.**
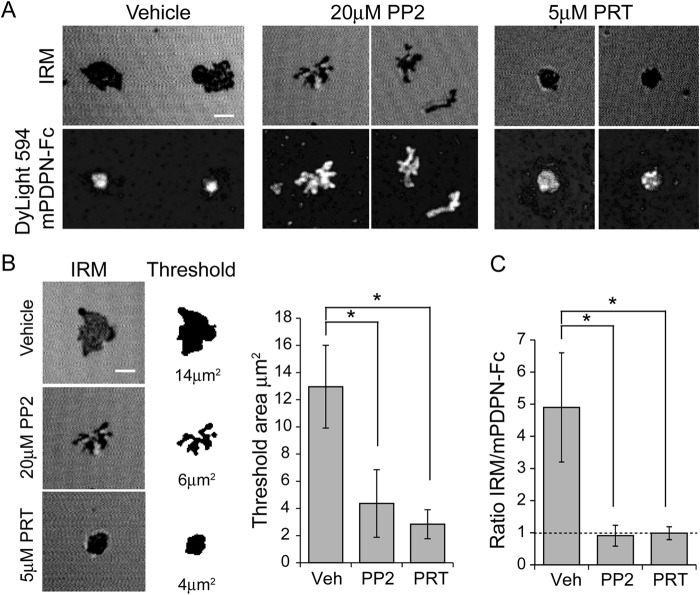
**Syk and Src family kinase inhibition decreases platelet-bilayer interactions.** *A*, platelets treated with vehicle, Syk (PRT-060318, 5 μm) or Src family (PP2 20 μm) kinase inhibitors were allowed to interact with glass-supported lipid bilayers containing Dylight 594-labeled mPDPN-Fc for 45 min at 37 °C. IRM and fluorescent images of the platelet/bilayer interface. *Top panels*, IRM; *bottom panels*, confocal images of Dylight 594-labeled mPDPN-Fc. *B*, example platelet contact area shown as the original unthresholded image (*left panels*) or after thresholding and binarization (*right panels*). Quantification of close surface contact areas of platelets interacting with mobile Podoplanin is shown. *C*, the ratio of the IRM threshold area to mPDPN-Fc area. The results are the averages of three separate experiments ± S.D. *, *p* < 0.05. *Veh*, vehicle.

Because the reduction in adhesion upon kinase inhibition was not due to a decrease in platelet spreading, we next considered the possibility that it was caused by a reduction in the platelet-Podoplanin contact area. We used IRM to address this and compared the results to the localization of labeled mPDPN-Fc tethered to the supported lipid bilayers (Fig. 4). Destructive interference between the phase-shifted reflections from the glass to media interface and in the phase reflections from the media-cell interface results in dark areas when the separation of the cell from the glass substrate is less than half the wavelength of the incident light. Dark areas indicate areas of close platelet-substrate contact.

Vehicle-treated platelets form a contact area similar to the platelet area with evidence of dynamic lamellipodial extension and collapse at the margins with a high degree of overall circularity; in contrast, the fluorescent mPDPN-Fc forms a central single bright cluster filling the center of the contact area resulting in an IRM/mPDPN-Fc ratio greater than 1 (Fig. 4, *A* and *C*). Inhibition of Src family kinases with PP2 resulted in a reduction in the overall close contact area with low circularity. Finger like projections were entirely filled with labeled mPDPN-Fc, resulting in an IRM/mPDPN-Fc ratio of ∼1, whereas the inhibition of Syk with PRT-060318 resulted in a highly circular contact entirely filled with labeled mPDPN-Fc and an IRM/mPDPN-Fc ratio of ∼1 (Fig. 4, *A–C*). These results demonstrate that the role of Src and Syk kinases in supporting platelet adhesion to the supported lipid bilayers is mediated through regulation of the area of contact in distinct ways. The differential structures seen in the presence of inhibitors of the two kinases is likely because Src family kinases are upstream of Syk kinases, which may have scaffolding roles in the absence of kinase activity, or because Src kinases have additional substrates besides Syk downstream of CLEC-2 activation.

##### Syk and Src Family Kinase Inhibition Alters Podoplanin Cluster Dynamics

We performed live cell imaging to investigate the dynamics of platelet-mediated clustering of Podoplanin. This was achieved by monitoring the interaction of Lifeact-GFP-expressing platelets with Dylight 594-labeled Podoplanin in a supported lipid bilayer using TIRF microscopy. Lifeact-GFP is a specific probe for the actin cytoskeleton that enables live cell monitoring of cytoskeletal rearrangements (31) and can be used to image the interplay between platelet cytoskeletal rearrangements and Podoplanin localization.

**FIGURE 5.**
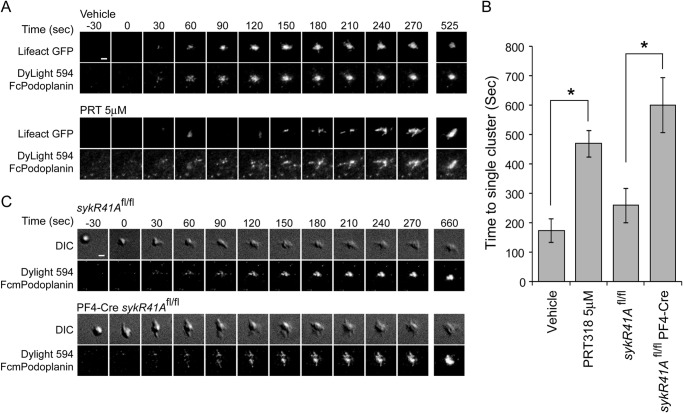
**Role of Syk kinase in platelet-mediated Podoplanin cluster dynamics.** *A*, TIRFM time course of Lifeact-GFP-expressing platelets interacting with planar lipid bilayer containing Dylight 594-labeled mPDPN-Fc. The *top panels* show control Lifeact-GFP-expressing platelets. The *bottom panels* show Lifeact-GFP platelets in the presence of a Syk kinase inhibitor (PRT-060318, 5 μm; see supplemental Movie S1). *B*, quantification of the time taken to form a single stable central structure from the first point of contact. The results are the averages of three separate experiments ± S.D. *, *p* < 0.01. *C*, DIC time course of control (*sykR41A*^fl/fl^; *upper panels*) or SykR41A expressing (PR4-Cre*sykR41A*^fl/fl^; *lower panels*) platelets interacting with planar lipid bilayer containing Dylight 594-labeled mPDPN-Fc (see supplemental Movie S2). Time is relative to the first detected contact point. *Scale bars*, 2 μm.

As soon as the platelet contacts the membrane, Podoplanin accumulation under the platelet can be detected. Initially small clusters of Podoplanin form that rapidly migrate with a centripetal motion to form a bright central cluster (Fig. 5*A*, *upper panels*, and supplemental Movie S1). The robust formation of a central cluster occurs within 4 min (Fig. 5*B*).

The role of Syk in Podoplanin cluster dynamics was then monitored in the presence of a Syk inhibitor, 5 μm PRT-060318 (Fig. 5*A*, *lower panels*, and supplemental Movie S1). Syk inhibition impeded the formation of the central Podoplanin cluster and thereby increased the time to cluster formation to over 8 min (Fig. 5*B*). To validate the results using the Syk inhibitor, we also monitored the interaction of platelets expressing SykR41A with bilayers containing Dylight 594-labeled Podoplanin. As seen with the Syk inhibitor, the dynamics of platelet-mediated Podoplanin clustering were impeded in the presence of platelets expressing SykR41A (Fig. 5*C* and supplemental Movie S2). When compared with control platelets, there was a delay in the formation of a central Podoplanin structure, which was similar to platelets treated with the Syk inhibitor PRT-060318 (Fig. 5*B*). These data suggest that the clustering of Podoplanin is dependent on the binding of Syk to phosphotyrosine and on its kinase activity.

**FIGURE 6.**
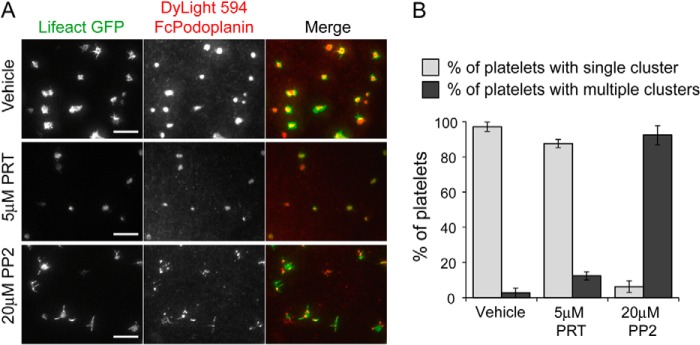
**Src family kinase inhibition blocks Podoplanin cluster dynamics.** *A*, TIRFM images of Lifeact-GFP-expressing platelets interacting with planar lipid bilayer containing Dylight 594-labeled mPDPN-Fc for 45 min at 37 °C. Lifeact-GFP-expressing platelets were pretreated with Src family kinase inhibitor (PP2, 20 μm) or a Syk inhibitor (PRT-060318, 5 μm). *Scale bar*, 10 μm. *B*, quantification of the cluster morphology in the presence and absence of Src family (PP2, 20 μm) or a Syk (PRT-060318, 5 μm) kinase inhibitors at 45 min. Platelets were identified as having a single or multiple Podoplanin clusters. Images and results are the averages of three independent experiments ± S.D.

We further investigated whether platelet-mediated formation of Podoplanin clusters was dependent on Src family kinases using the Src family kinase inhibitor, PP2. In the presence of 20 μm PP2, platelets formed numerous Podoplanin clusters, but these clusters rarely coalesced to form a large central structure within 45 min, as seen in control platelets and in platelets treated with the Syk inhibitor PRT-060318 (Fig. 6). Thus, the defect in the formation of a large central structure in the absence of Src family kinase activity is more severe than the clustering defect in the absence of Syk activity. This is consistent with Syk contributing to central clustering independent of its catalytic activity or phosphotyrosine binding of its N-terminal SH2 domain and with Src family kinase activity being necessary for Syk to have this role.

##### Syk or Src Family Kinase Inhibition Decreases Tight Podoplanin Clustering

The above results show that the inhibition of the tyrosine kinases downstream of CLEC-2 results in changes in Podoplanin cluster formation by platelets. Although TIRF imaging permits us to visualize cluster formation, it does not have sufficient resolution to allow us to determine the proximity of Podoplanin molecules to one another. To determine whether platelets cluster Podoplanin molecules within 10 nm of one another, we used FLIM to detect FRET (FRET-FLIM) between Podoplanin molecules in the planar bilayer.

**FIGURE 7.**
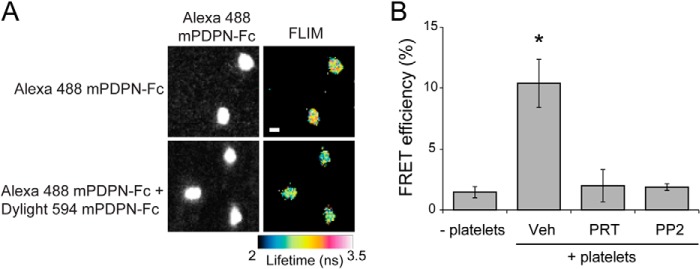
**Syk and Src family kinase inhibition decreases Podoplanin clustering.** *A*, platelets interacting with planar lipid bilayer containing Alexa 488-labeled mPDPN-Fc or equivalent amounts of Alexa 488-labeled mPDPN-Fc and Dylight 594-labeled mPDPN-Fc for 45 min at 37 °C. Fluorescence lifetimes of the donor fluorophore (mPDPN-Fc-488) were determined in the absence and presence of acceptor fluorophore (mPDPN-Fc-594). The fluorescence lifetimes in nanoseconds (ns) are shown as false color images. *Scale bar*, 2 μm. *B*, FRET-FLIM analysis of mPDPN-Fc containing bilayers incubated with platelets treated with or without inhibitors to Src family (PP2, 20 μm) or Syk (PRT-060318, 5 μm) kinases. Images and results are the averages of three independent experiments ± S.D. *Veh*, vehicle.

To enable FRET-FLIM measurements, mPDPN-Fc was conjugated to Alexa Fluor 488 (donor: 488 mPDPN-Fc) or Dylight 594 (acceptor: 594 mPDPN-Fc). Platelets were allowed to interact with bilayers containing 488 mPDPN-Fc or equivalent levels of donor 488 mPDPN-Fc and acceptor 594 mPDPN-Fc for 45 min. Samples were fixed, and the average lifetime of 488 mPDPN-Fc was determined in the absence and presence of the acceptor 594 mPDPN-Fc for platelets incubated with vehicle, Syk inhibitor, PRT-060318, or Src family kinase inhibitor, PP2. Fluorescence lifetimes were used to calculate the FRET efficiency. In the presence of vehicle, platelets showed a decrease in donor fluorescence lifetime, resulting in a FRET efficiency of 10 ± 2%. This indicates that the distance of Podoplanin molecules was within 10 nm of one another (Fig. 7) and that platelets promote the formation of tight Podoplanin clusters. Inhibition of Syk and Src family kinases results in a loss of FRET, indicating that the average distance between Podoplanin molecules within the bilayer was greater than 10 nm.

##### Platelets Cluster Podoplanin in HEK293T Cells

**FIGURE 8.**
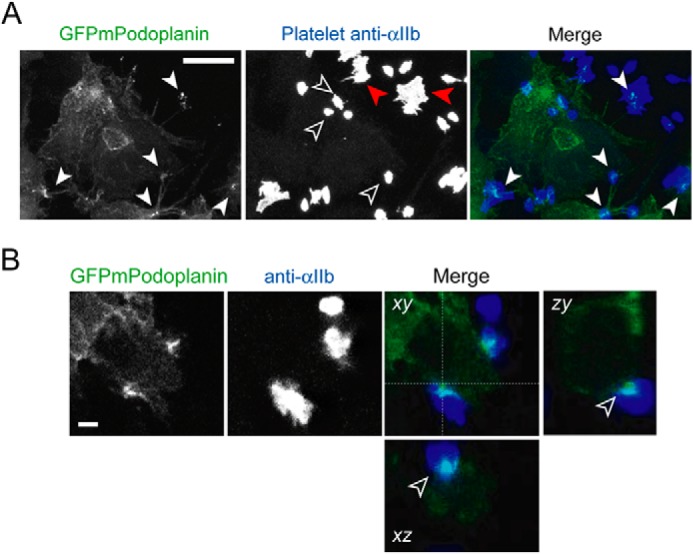
**Podoplanin is recruited at the platelet-cell interface.** *A*, HEK293T cells expressing GFPmPodoplanin were analyzed in the presence of platelets by confocal microscopy. Maximum intensity projection of GFPmPodoplanin HEK293T cells (*left panel*) and platelets stained with an anti-αIIb antibody (*middle panel*). The merged images are shown in the *right panel* (GFPmPodoplanin, *green*; anti-αIIb, *blue*). Clusters of GFPmPodoplanin associated with a platelet are indicated by *closed white arrowheads* in the GFPmPodoplanin image and superimposed on the merge image. Platelets only interacting with the GFPmPodoplanin HEK293T cell are indicated by *open arrowheads*. Platelets interacting with both the GFPmPodoplanin HEK293T cell and the coverslip are indicated by *red arrowheads. Scale bar*, 10 μm. *B*, confocal image of GFPmPodoplanin (*left panel*) and anti-αIIb antibody (*middle panel*). The merged images are shown in the *right panel* (GFPmPodoplanin, *green*; anti-αIIb, *Blue*). *xz* and *yz* projections display a cross-section of a cell-platelet interaction. The accumulation of GFPmPodoplanin between the cell and the platelet is indicated by an *open arrowhead* in the *xz* and *yz* views. *Scale bar*, 2 μm. Images are representative of three independent experiments.

Thus far, our results demonstrate that platelets are able to cluster Podoplanin contained within a supported lipid bilayer. To examine whether platelets are able to cluster Podoplanin on an intact cell surface, we analyzed the interaction of platelets with HEK293T cells overexpressing a C-terminal green fluorescent protein from *Aequorea coerulescens* (AcGFP) fusion of mouse Podoplanin (mPodoplanin). Following the interaction of platelets with the AcGFPmPodoplanin-expressing HEK293T cells for 45 min, the cells were fixed, and the platelets were identified using an antibody against the integrin subunit αIIb. Despite the fact that HEK293T cells express significant levels of endogenous human Podoplanin, we found that very few mouse platelets interacted with control cells expressing GFP alone (data not shown). In contrast, HEK293T cells expressing mouse Podoplanin support mouse platelet adhesion. The platelets interacting solely with the AcGFPmPodoplanin-expressing HEK293T cells show a reduced level of spreading when compared with platelets that interact with both the cell and the coverslip (Fig. 8*A*). Interestingly, confocal microscopy revealed a local accumulation of Podoplanin below an attached platelet, which is occurring at the interface between individual platelets and AcGFPmPodoplanin-expressing cells (Fig. 8, *A* and *B*).

**FIGURE 9.**
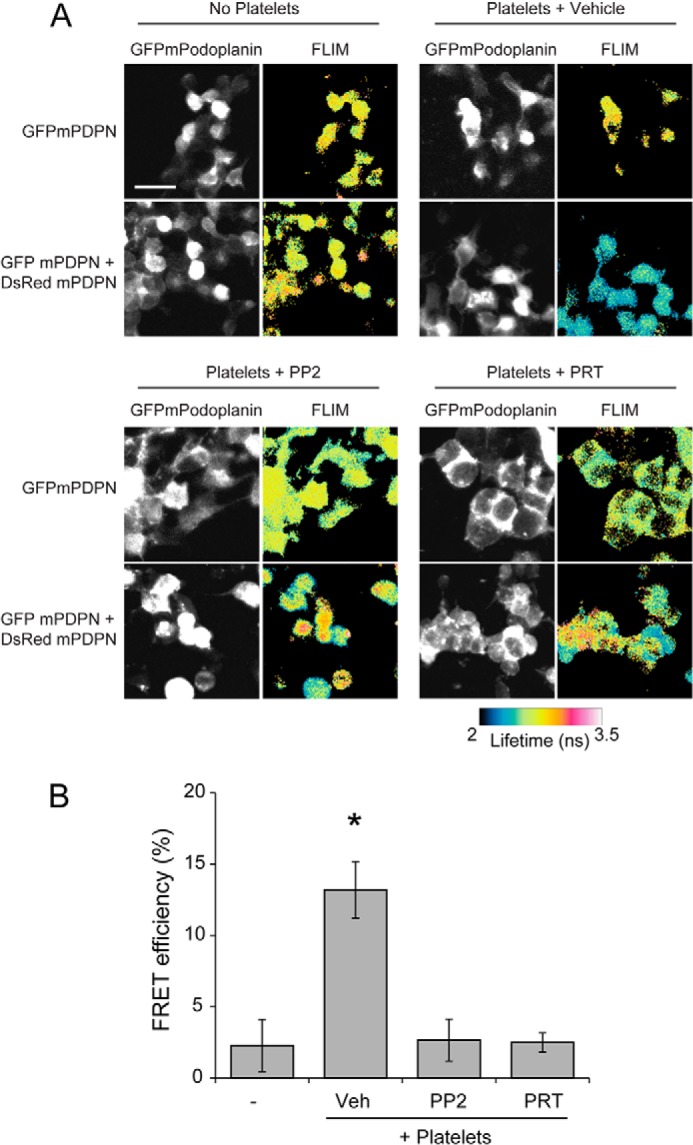
**CLEC-2 signaling leads to Podoplanin clustering in a cell membrane.** *A*, platelets were allowed to interact with cells expressing either AcGFPmPodoplanin or cells expressing equivalent levels of AcGFPmPodoplanin and DsRedmonomer-mPodoplanin for 1 h. Samples were fixed, and the average lifetime of AcGFPmPodoplanin was determined in the absence and presence of the acceptor DsRedmPodoplanin for cells incubated with platelets treated with vehicle, Syk (PRT-060318, 5 μm), or Src family (PP2 20 μm) kinase inhibitors. The fluorescence lifetimes in nanoseconds (ns) are shown as false color images. *Scale bar*, 10 μm. *B*, FRET-FLIM analysis of cells expressing AcGFPmPodoplanin incubated with platelets incubated with or without Syk (PRT-060318, 5 μm) or Src family (PP2 20 μm) kinase inhibitors. Images and results are the averages of three separate experiments ± S.D. *, *p* < 0.01. *Veh*, vehicle.

To assess clustering more quantitatively, we performed FRET-FLIM on cells expressing either AcGFPmPodoplanin or equivalent amounts of AcGFPmPodoplanin and DsRedmonomer-mPodoplanin (Fig. 9*A*). Platelets were allowed to interact with cells expressing either AcGFPmPodoplanin or cells expressing equivalent levels of AcGFPmPodoplanin and DsRedmonomer-mPodoplanin for 1 h. Samples were fixed, and the average lifetime of AcGFPmPodoplanin was determined in the absence and presence of the acceptor DsRedmPodoplanin for cells incubated with platelets treated with vehicle, Syk inhibitor, PRT-060318, or Src family kinase inhibitor, PP2. Fluorescence lifetimes were subsequently used to calculate the FRET efficiency (Fig. 9*B*).

In the absence of platelets, no FRET was observed (Fig. 9*B*). Cells incubated with platelets in the presence of vehicle showed a decrease in fluorescence lifetime, resulting in a FRET efficiency of 13 ± 2%. This indicates that the distance between Podoplanin molecules was within 10 nm, suggesting that platelets can initiate the formation of tight clusters of Podoplanin in cells. Inhibition of Syk and Src family kinases results in a loss of FRET. These results agree with those on the interaction of platelets with Podoplanin in an artificial bilayer and thereby demonstrate a critical role for CLEC-2 signaling in supporting adhesion to Podoplanin on a cell surface.

##### CLEC-2 Forms Clusters When Exposed to a CLEC-2 Ligand

**FIGURE 10.**
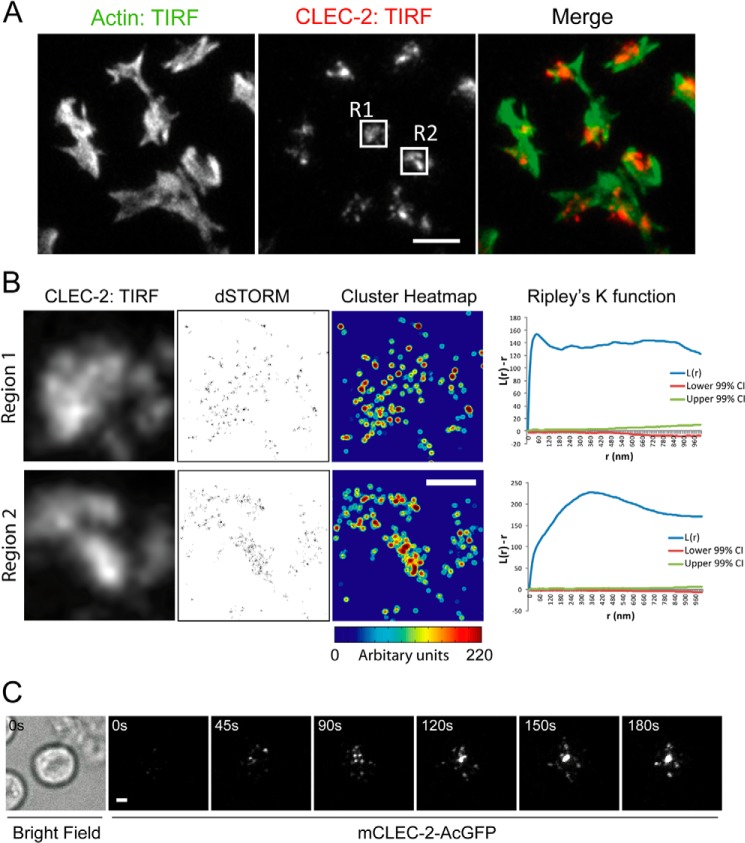
**CLEC-2 can form clusters that, following ligand engagement, migrate in a directed manner toward the center of the cell.** *A*, washed mouse platelets were allowed to interact with mPDPN-Fc (10 μg/ml)-coated glass dishes for 45 min at 37 °C. TIRFM image of Alexa 488-phalloidin (*left panel*) and Alexa 647-anti-CLEC-2 (INU1) (*middle panel*). The merged images are shown in the *right panel* (actin, *green*; mCLEC-2, *red*). Two regions of interest are highlighted, *R1* and *R2. Scale bar*, 5 μm. *B*, TIRFM images of Alexa 647-anti-mCLEC-2 in the *boxed regions* from Fig. 10*A* (R1 and R2; *left panels*). dSTORM images of regions 1 and 2 (*middle panels*). Quantitative cluster mapping of the dSTORM images of regions 1 and 2 (*right panels*). *Scale bar*, 1 μm. Ripley's K-function analysis of the molecules in regions 1 and 2; *L*(*r*) − *r* reports the degree of clustering relative to a random distribution (indicated by the lower and upper 99% confidence intervals (*CI*) (*red* and *green lines*)), and *r* is the radial scale. *C*, TIRFM time course of a mCLEC-2-AcGFP-expressing DT40 chicken B cell interacting with a planar lipid bilayer containing an CLEC-2-activating antibody (17D9) (see supplemental Movie S3). The bright field image shows the location of the cell. *Scale bar*, 2 μm. Images are representative of three independent experiments.

We infer that CLEC-2 forms clusters by investigating the distribution of its ligand Podoplanin. To investigate the distribution of CLEC-2 directly, mouse platelets were spread on recombinant Podoplanin. The actin cytoskeleton was stained with Alexa 488-phalloidin, and CLEC-2 was stained with Alexa 647-anti-mCLEC-2 antibody (INU1). TIRF imaging demonstrates that CLEC-2 forms large clusters when platelets interact with Podoplanin (Fig. 10*A*). To further resolve the spatial distribution of CLEC-2, we used dSTORM. This super resolution method is able to resolve proteins with an ∼20-nm spatial resolution (Fig 10*B*). To analyze the distribution of CLEC-2 molecules, we used a modified quantitative cluster mapping, which has previously been used to analyze the distribution of proteins molecules using dSTORM data (40). Molecules that display an increased cluster distribution are presented as a cluster heat map with “hot” colors identifying highly clustered molecules. (Fig. 10*B*). The analysis demonstrates that the large cluster observed using TIRFM is composed of smaller nanoscale clusters. To assess the degree of clustering, we analyzed the distribution of CLEC-2 by Ripley's K function (Fig. 10*B*). Ripley's K-function analysis of the molecules in regions 1 and 2 reports a significant increase in the degree of clustering relative to a random distribution (indicated by the 99% confidence intervals). Overall, we show that CLEC-2 is highly clustered when platelets interact with recombinant Podoplanin.

To investigate both the spatial and temporal distribution of CLEC-2, we generated an N-terminal GFP fusion of mouse CLEC-2, which maintained receptor function when expressed in a DT40 chicken B cell line (data not shown). Cells expressing mCLEC-2-GFP were allowed to interact with a supported lipid bilayer containing an activating antibody to CLEC-2 (17D9) and imaged using TIRF microscopy. Following ligand engagement, macroclusters of CLEC-2 migrate in a directed manner toward the center of the cell (Fig. 10*C* and supplemental Movie S3). This is comparable with the distribution of Podoplanin in a supported lipid bilayer by platelets.

## DISCUSSION

In this study, we have shown that CLEC-2 on platelets induces the dynamic formation of tight clusters of its ligand Podoplanin in a mechanism that is dependent on Src family and Syk kinase activity. Moreover, we demonstrate that the clustering mechanism greatly enhanced platelet adhesion to Podoplanin. In the presence of inhibitors of these kinases, Podoplanin molecules are no longer tightly clustered because FRET efficiency between donor-labeled and acceptor-labeled Podoplanin molecules is negligible. The individual binding affinity of Podoplanin for CLEC-2 is low for an interaction that can support cell adhesion (*K_d_* = 24 μm) (11). We hypothesize that Podoplanin clustering by platelets leads to an increase in avidity, which augments cell adhesion. The increase in avidity is dependent on Src family and Syk kinase activation in platelets and is likely mediated by centralization of CLEC-2 receptors into a cluster, thereby also clustering Podoplanin, as demonstrated in the supported lipid bilayer model.

Many single spanning membrane receptors do not function as individual signaling units but instead associate in multimolecular complexes. Formation of submicron clusters to initiate signaling followed by consolidation in a larger central cluster (macrocluster) is characteristic of ITAM/hemITAM containing immunoreceptors. The formation of a large central cluster on this scale enhances the intracellular signal and has the advantage of restricting the diffusion of molecules at the membrane, such as the exclusion of membrane associated negative regulators like CD45 (41, 42). Larger scale clustering can also be associated with signal termination (43), maintenance (44), or enhancement (45) in different systems. There is now considerable evidence to indicate that the size and spatial distribution of signaling assemblies are highly regulated. Receptor redistribution can result in dynamic regulation of signaling from its initiation to down-regulation. In the present study, we show that CLEC-2-mediated signaling by Src family and Syk kinases plays a key role in the formation of large Podoplanin (macro)clusters in a Podoplanin-containing supported lipid bilayer and in a cell membrane.

The stoichiometry of Podoplanin and CLEC-2 has been reported to be one molecule of Podoplanin per CLEC-2 dimer (46). Thus, dimerization of CLEC-2 in the absence of higher order clustering is not expected to bring Podoplanin monomers within 10 nm of each other. Therefore, the potential requirement of Src and Syk kinase activity and Syk SH2 function to form CLEC-2 dimers is not sufficient to account for the induced Podoplanin proximity as measured by FLIM and must reflect higher order clustering. At the same time, the formation of a central, albeit enlarged cluster is not sufficient to induce the molecular scale Podoplanin clustering because Syk inhibition and the Syk SH2 mutant eventually form a central cluster but still lack the FRET signal indicative of bringing Podoplanin monomers within 10 nm of each other.

The use of the supported lipid bilayer system revealed an unexpected difference between the roles of Src family kinase and Syk activity in cluster transport. Src inhibition resulted in a more severe phenotype in which central clustering was inhibited, whereas Syk inhibition resulted in impeded central clustering. This result may reflect the potential for Syk to act as a Src family kinase-dependent scaffold protein linking CLEC-2 clusters to a cell-efficient centralization process or the possible activation of additional substrates by the Src kinase. A scaffolding role for Syk family kinases has been proposed previously based on binding of adapters to the interdomain phosphotyrosines, which are phosphorylated in part by Src family kinases (47).

The present results are similar to the formation of the immunological synapse that underlies signaling by the ITAM containing T and B cell receptors (33, 48, 49). However, a notable difference is the reduced dependence of ITAM receptors on Src family kinases in mediating clustering (49–52), which may reflect a differential role of the ITAM and hemITAM signaling units. In this context, Src and Syk family kinase-mediated formation of the CLEC-2 dimer may be a necessary step to higher order clustering of the ligand, whereas this is not the case for an ITAM receptor.

The molecular basis of the requirement for platelet signaling during lymphatic development is not known, although it does not appear to depend on integrin activation or dense/α-granule secretion (17, 53, 54). Interestingly, we and others have shown that addition of platelets to LECs leads to a decrease in endothelial cell migration *in vitro* and that this is mirrored by the clustering of Podoplanin by cross-linking antibodies or recombinant CLEC-2 (17, 18). A similar decrease in migration is seen on antibody cross-linking of Podoplanin in osteosarcoma cells (55). There is considerable evidence of a role for Podoplanin in regulating cell migration. Up-regulation and knockdown of Podoplanin has been reported to increase and decrease cell migration, respectively (13, 16). These ligand-independent effects may reflect constitutive signaling by Podoplanin. Thus, inhibition of Podoplanin-regulated migration as a consequence of interaction with CLEC-2- and Syk-dependent platelet activation provides evidence for an attractive model to explain how platelet adhesion serves to halt migration of lymphatic vessels upon contact with blood endothelial cells and thus prevent joining of the two vasculatures during embryonic development. This model is particularly attractive given the recent demonstrations using ultramicroscopy that LECs migrate away from the cardinal vein as single cells and only later on express Podoplanin and join together to form lymphatic sacs and lymphatic vessels (56). The inhibition of migration by platelets when in contact with blood endothelial cells would prevent joining of the two vasculatures.

Our results also raise the possibility that stable adhesion between platelets and LECs contributes to the prevention of blood-lymphatic mixing. Podoplanin clustering by CLEC-2-mediated signaling *in vivo* is likely to contribute to the binding of platelets to LECs. Additionally we have shown that CLEC-2 signaling influences the degree of Podoplanin clustering on the LEC surface. We therefore speculate that, in a similar manner to the effect that cross-linking of Podoplanin by specific antibodies or recombinant CLEC-2 has on LEC migration (17, 18), CLEC-2-mediated signaling regulates cross-linking of Podoplanin molecules and thereby influences the motility of LECs and thus halts migration upon contact with blood endothelial cells. Podoplanin depletion causes Cdc42 activation and RhoA inhibition, resulting in a decrease in lung microvascular endothelial cell migration (16). It may be that formation of tight clusters of Podoplanin initiated by platelet CLEC-2-mediated signaling can regulate the activity of small GTPases, which leads to inhibition of LEC migration.

Our results have implications for other physiologically relevant interactions between platelet CLEC-2- and Podoplanin-expressing cells. It has been recently reported that CLEC-2 plays a role in the prevention of inflammation-induced hemorrhage (57). Additionally, Podoplanin expressed on fibroblastic reticular cells surrounding high endothelial venules acts as an activating ligand for platelet CLEC-2 to release sphingosine 1-phosphate, which mediates up-regulation of VE-cadherin on the endothelial cells, thus preventing leakage of blood into the lymph node (58). Podoplanin up-regulated in metastatic tumors has been proposed to promote CLEC-2 platelet activation and aggregation. The interaction between CLEC-2 and Podoplanin has been associated with tumor cell arrest and extravasation (7, 59–61). Platelet-mediated clustering of Podoplanin may play a role in these processes by maintaining stable adhesion as well as by regulating the level of platelet activation and through inhibition of Podoplanin signaling.

CLEC-2 is also expressed on other hematopoietic lineages, including dendritic cells (1, 2). Our findings therefore have implications for the interaction of other CLEC-2-expressing cells with Podoplanin. One such example is the role of CLEC-2 in regulating the migration of dendritic cells to lymph nodes (62).

**FIGURE 11.**
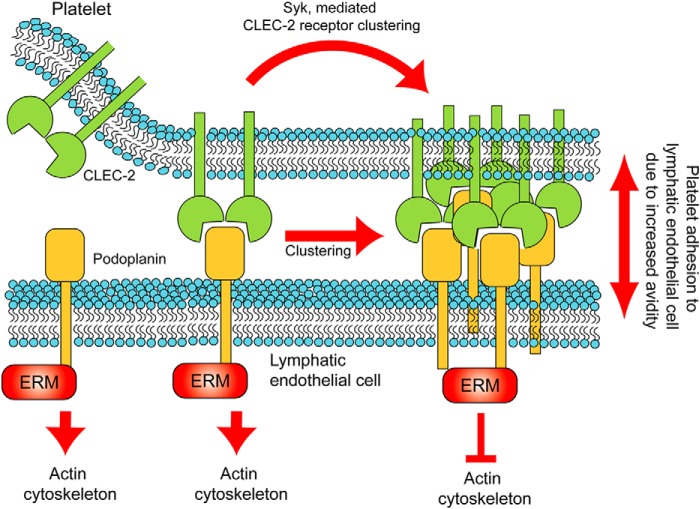
**Model summary of CLEC-2-mediated clustering of Podoplanin.** We propose that the binding of CLEC-2 to Podoplanin enhances the adhesion of platelets to lymphatic endothelial cells through CLEC-2-mediated receptor clustering involving the kinase Syk. We hypothesize that this would in turn lead to clustering of Podoplanin and functional effects in the LECs by modulation of Podoplanin signaling though ERM proteins and regulation of the actin cytoskeleton.

Our study has taken full advantage of the supported planar bilayer model to advance the understanding of platelet hemITAM signaling in maintaining adhesion. To our knowledge, this is the first time this model system has been used to provide mechanistic insight into platelet and hemITAM signaling. Our findings were supported by the use of cells expressing GFPmPodoplanin in which an accumulation of Podoplanin can be found at the interface between a platelet and a cell. We propose that the binding of CLEC-2 to Podoplanin enhances the adhesion of platelets to LECs through CLEC-2-mediated receptor clustering. We hypothesize that this would in turn lead to clustering of Podoplanin and functional effects in the LECs by down-regulation of Podoplanin signaling though ERM proteins and the small GTPases Cdc42 and RhoA (see the model in Fig. 11). These observations are likely to contribute to the biology of CLEC-2 and Podoplanin, including their role in prevention of blood-lymphatic mixing during development that has recently been proposed to occur at sites of low shear where the lymph is returned to the bloodstream in the thoracic duct (63), as well as through formation of blood-lymphatic contacts (18, 24, 38).

## Supplementary Material

Supplemental Data

## Footnotes

4 C. E. Hughes, L. Navarro-Nuñez, and S. P. Watson, unpublished data.

## Footnotes

3 The abbreviations used are:

CLEC-2: C-type lectin receptor 2
LEC: lymphatic endothelial cell
DIC: differential interference contrast
IRM: interference reflectance microscopy
TIRF: total internal reflection fluorescence
FLIM: fluorescence lifetime imaging
dSTORM: direct stochastic optical reconstruction microscopy
ITAM: immunoreceptor tyrosine-based activation motif.
