# Supplementary material for: Syk and Src Family Kinases Regulate C-type Lectin Receptor 2 (CLEC-2)-mediated Clustering of Podoplanin and Platelet Adhesion to Lymphatic Endothelial Cells
Source: J Biol Chem. 2014 Nov 3;289(52):35695–710. doi: 10.1074/jbc.M114.584284 (PMC4276840; doi:10.1074/jbc.M114.584284)
Supplement: Supplemental Data [file supp_289_52_35695__index.html]

Syk and Src Family Kinases Regulate C-type Lectin Receptor 2 (CLEC-2)-mediated Clustering of Podoplanin and Platelet Adhesion to Lymphatic Endothelial Cells — CLEC-2 Mediates Podoplanin Clustering — Supplemental Data 

# Syk and Src Family Kinases Regulate C-type Lectin Receptor 2 (CLEC-2)-mediated Clustering of Podoplanin and Platelet Adhesion to Lymphatic Endothelial Cells

## Supplemental Data

**Files in this Data Supplement:**

- Supplemental Movie 3  - SUPPLEMENTAL MOVIE 3. CLEC-2 can form microclusters which, following ligand engagement, migrate in a directed manner towards the centre of the cell. TIRFM time course of a mCLEC-2-GFP expressing DT40 chicken B cell interacting with a planar lipid bilayer containing and activating antibody (17D9). Frames were taken every 5 seconds. Scale bar 2?m.
- Supplemental Movie 2  - SUPPLEMENTAL MOVIE 2. Platelets expressing SykR41A display impeded dynamics of platelet-mediated Podoplanin clustering. DIC microscopy time course of control (SykR41A fl/fl; top left panel) or SykR41A expressing (PF4-CreSykR41Afl/fl; top right panel) platelets when interacting with lipid bilayer containing Dylight594 labelled mPDPN-Fc (Bottom panels). Frames were taken every 5 seconds.
- Supplemental Movie 1  - SUPPLEMENTAL MOVIE 1. Syk inhibition inhibits the dynamics of platelet-mediated Podoplanin clustering. TIRF microscopy time course of Lifeact-GFP expressing control platelets (left panels) or Syk inhibitor treated (PRT-060318, 5?M) (right panels) Lifeact-GFP platelets interacting with supported lipid bilayer containing Dylight594 labelled mPDPN-Fc (Bottom panels). Frames were taken every 5 seconds.
